# The association between immune cells and acute kidney injury: insights from Mendelian randomization

**DOI:** 10.1080/0886022X.2025.2471011

**Published:** 2025-03-02

**Authors:** Xianzhen Yang, XiaoLei Zhang, Denglu Zhang, Shengtian Zhao

**Affiliations:** a Department of Urology, Shandong Provincial Hospital, Shandong University, Jinan, China; b Department of Urology, Affliated Hospital of Shandong University of Traditional Chinese Medicine, Jinan, China; c Department of Clinical Laboratory, Jinan Fourth People’s Hospital, Jinan, China; d Central Laboratory, Affiliated Hospital of Shandong University of Traditional Chinese Medicine, Jinan, China; e Shandong Key Laboratory of Dominant Diseases of traditional Chinese Medicine, Affiliated Hospital of Shandong University of Traditional Chinese Medicine, Jinan, China; f Department of Urology, Qilu Hospital of Shandong University, Jinan, China

**Keywords:** Immunopheno types, acute kidney injury, rapid kidney function decline, mendelian randomization analysis, genetic analyses

## Abstract

**Background:**

Immune disorder is a prominent feature of acute kidney injury (AKI). However, the specific role of different immune cell phenotypes in the pathogenesis of AKI remains poorly understood. The aim of this study was to investigate the relevance of 731 immune phenotypes to AKI.

**Materials and Methods:**

We obtained data on 731 immune cell phenotypes from the GWAS Catalog and Open GWAS databases and undertook a series of quality control measures to identify exposure-related instrumental variables (IV). Data on acute renal failure (ARF) and rapid kidney function decline were obtained from the Finngen R11 and CKDGen databases as an outcome. Subsequently, we performed two-sample Mendelian randomization (MR) using inverse variance weights to explore the causal relationship between 731 immune cell characteristics and ARF and rapid kidney function declineas at the gene level. Sensitivity analyses, including leave-one-out and other MR analysis models, were performed.

**Results:**

At the significance level corrected by Bonferroni, 9 immune phenotypes, including CD25 on IgD + CD24- B cell, were associated with ARF; 4 immune phenotypes, including SSC-A on plasmacytoid dendritic cell, were associated with rapid kidney function decline. Sensitivity analysis indicated robust results.

**Conclusion:**

Our study demonstrates a strong causal relationship between specific types of immune cells and AKI by genetic means, providing valuable insights for future clinical studies.

## Introduction

Acute kidney injury (AKI) is a significant clinical condition characterized by an abrupt reduction in kidney function, which can lead to severe complications such as chronic kidney disease (CKD) and increased mortality [1,2]. he global incidence of AKI is notably high, affecting approximately 10–15% of hospitalized patients, and is even more prevalent among patients in intensive care units, where the incidence exceeds 50% [3,4]. AKI is associated with a poor prognosis and an increased risk of developing end-stage renal disease, thereby imposing a substantial burden on healthcare systems.

There is growing evidence that immune cells are associated with AKI, including neutrophils, dendritic cells, macrophages and lymphocytes [5]. Notably, AKI is common in patients receiving immunotherapy, with one study showing that 25% of patients receiving immunotherapy developed AKI within one year [6]. In addition, a meta-analysis showed a 5.7% incidence of AKI and a median latency of 108.07 days in cancer patients treated with immunosuppressive agents [7]. The body’s immune system strictly controls tissue homeostasis and immune defense, and immune disruption is an important process in AKI, in which different types of immune cells play different roles.

**Figure 1.**
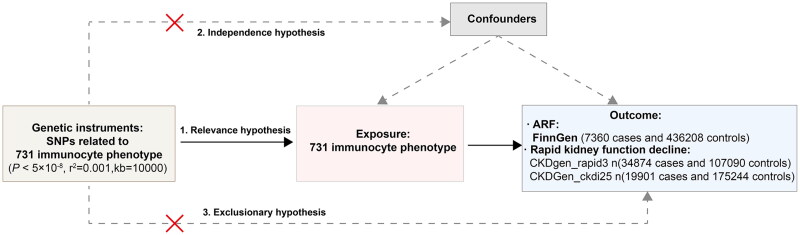
Framework for evaluating the causal impact of immunocyte phenotype on ARF.

Therefore, a more comprehensive understanding of the causal relationships between immune cell phenotypes and AKI is necessary to identify potential biomarkers and therapeutic targets. Mendelian randomization (MR) is a robust epidemiological approach that uses genetic variants as instrumental variables (IVs) to infer causality between an exposure (in this case, immune cell phenotypes) and an outcome (such as AKI) [8,9]. In the past three years, MR research in nephrology has grown rapidly and has gradually established standardized methodologies [10]. By leveraging genetic data, MR analysis can overcome many of the confounding biases that often limit observational studies, providing more reliable evidence for causal relationships [11]. In this study, we employed a two-sample MR approach to investigate the causal impact of 731 immune phenotypes on AKI, focusing specifically on acute renal failure (ARF) and rapid kidney function decline. Our objective was to identify immune phenotypes that could serve as potential biomarkers or therapeutic targets for AKI, thereby paving the way for novel intervention strategies. The flow chart of this study is shown in Figure 1.

## Materials and methods

### Study design

This study was conducted in accordance with the Strengthening the Reporting of Observational Studies in Epidemiology Using Mendelian Randomization (STROBE-MR) guidelines to ensure transparency and reproducibility [9]. We utilized summary data from published genome-wide association studies (GWAs) covering 731 immune cell features and ARF and rapid kidney function decline, and selected appropriate single nucleotide polymorphisms (SNPs) as instrumental variables (IVs) for MR analysis to explore causal relationships between them.

### GWAS data sources

Data on immune cell phenotypes were obtained from the GWAS Catalog, covering a total of 731 immune cell characteristics derived from a cohort of 3,757 individuals of European ancestry [12]. Outcome data on ARF and rapid kidney function decline were sourced from two independent databases: the FinnGen R11 database (https://r11.finngen.fi/), which included 443,568 individuals with 7,360 cases of ARF and 436,208 controls, and the CKDGen Consortium (https://ckdgen.imbi.uni-freiburg.de/datasets), which provided data on rapid kidney function decline [13]. All data were publicly available, and ethical approval was not required for the secondary analysis of these datasets. The sources of the above exposure and outcome data and sample information are shown in Table 1.

**Table 1.** Information on the GWAS data cohort used to conduct the MR analysis.

	Data source	Population	Phenotype	Sample size	
				Cases	Controls
Exposure	Orrù et al. (2020)	European	731 Immunophenotypes	3,757	
Outcome	FinnGen R11	European	Acute renal failure	7,360	436,208
	CKDGen	European	CKDi25	19,901	175,244
			Rapid3	34,874	107,090

### Instrumental variable selection

IVs must satisfy the three core assumptions of MR: they must be strongly associated with the exposure, independent of any confounding factors, and influence the outcome only through the exposure pathway (with no direct effects or pleiotropy) [14]. According to recent study, the selection criteria for single nucleotide polymorphisms (SNPs) are as follows [15]. IVs were selected based on SNPs significantly associated with 731 immune cell phenotypes at a genome-wide significance level (*p* < 5 × 10^−8^). To minimize the influence of linkage disequilibrium (LD), SNPs in LD (r^2^ > 0.001) within a 10,000 kb window around the most significant SNP were removed. The minor allele frequency (MAF) threshold of the variants of interest was 0.01. Additionally, weak instruments were excluded by setting an F-statistic threshold >10, calculated as F = (R^2^/(1 – R^2^)) * ((N – K − 1)/K).

### Statistical analysis

The primary analysis was conducted using the inverse variance-weighted (IVW) method, which provides an unbiased estimate of the causal effect when all IVs satisfy the core assumptions of MR. Additionally, we employed complementary MR methods, including MR-Egger regression, weighted median, and weighted mode, to assess the robustness of our findings and account for potential pleiotropy [16]. Heterogeneity among the selected IVs was assessed using Cochran’s Q test, and horizontal pleiotropy was evaluated using the MR-Egger intercept test [17,18]. To address potential outliers, we conducted leave-one-out sensitivity analyses, which involved systematically removing each SNP to examine its influence on the overall results [19].

All statistical analyses were performed using R software version 4.3.1, with the MendelianRandomization and TwoSampleMR packages. A Bonferroni correction was applied to adjust for multiple testing, with a significance threshold set at *p* < 0.0167 (0.05/3 outcomes) to minimize the likelihood of false-positive results.

## Results

First, after applying a series of quality control steps, we selected 1,892 SNPs as IVs from the 731 immune cell-associated SNPs. Detailed SNP information is provided in Supplementary document 1. As the primary analytical method, we used two-sample MR analysis and IVW methods to investigate the causal relationship between immune phenotypes and AKI. After bonferroni correction for *P*-values showed that 9 immune cell phenotypes were associated with ARF and four immune cell phenotypes were associated with rapid kidney function decline.

### Association between immune phenotypes and acute renal failure

**Figure 2.**
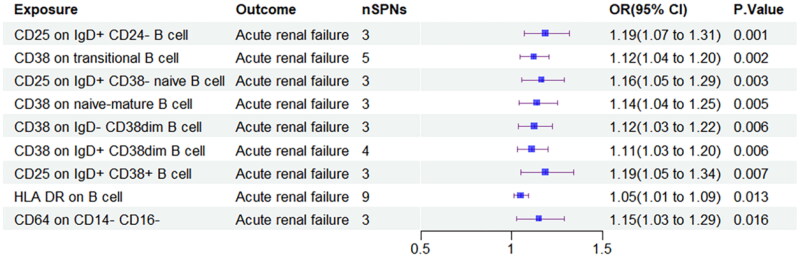
Forest plot of positive results in MR analysis of the role of immunophenotypes on ARF.

After applying the Bonferroni correction for multiple comparisons, 9 immune cell phenotypes were found to be significantly associated with ARF. These included CD25 on IgD + CD24- B cells (OR = 1.186, 95% CI = 1.070–1.314, *p* = 0.001), CD38 on transitional B cells (OR = 1.121, 95% CI = 1.044–1.203, *p* = 0.002), and CD25 on IgD + CD38- naive B cells (OR = 1.164, 95% CI = 1.053–1.287, *p* = 0.003). Other phenotypes included CD38 on naive-mature B cells, and HLA DR on B cells, each showing a statistically significant association with increased ARF risk (Figure 2). CD38 on various B cell subtypes, including IgD- CD38dim and IgD + CD38dim B cells, also showed positive correlations with ARF risk, suggesting a role for CD38-mediated signaling pathways in the progression of renal injury. Notably, CD64 on CD14- CD16- monocytes was also associated with ARF (OR = 1.150, 95% CI = 1.027–1.289, *p* = 0.016), indicating a potential link between monocyte activation and kidney damage.

### Association between immune phenotypes and rapid kidney function decline

**Figure 3.**
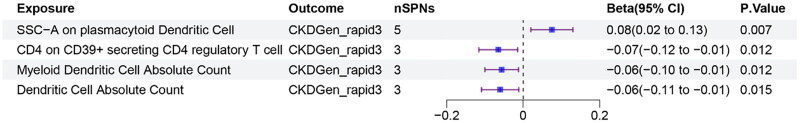
Forest plot of positive results in MR analysis of the role of immunophenotypes on rapid kidney function decline.

Four immune phenotypes were identified as significantly associated with rapid kidney function decline (Figure 3). SSC-A on plasmacytoid dendritic cells (pDCs) was positively associated with rapid kidney function decline (β = 0.08, 95% CI = 0.020–0.130, *p* = 0.007). In contrast, CD4 on CD39+ secreting regulatory T cells (β = −0.065, 95% CI = −0.116–-0.014, *p* = 0.012), myeloid dendritic cell absolute count (β = −0.056, 95% CI = −0.100–-0.012, *p* = 0.012), and dendritic cell absolute count (β = −0.060, 95% CI = −0.109–-0.012, *p* = 0.015) were negatively associated with rapid kidney function decline, suggesting potential protective roles for these cell types.

### Sensitivity analyses

**Figure 4.**
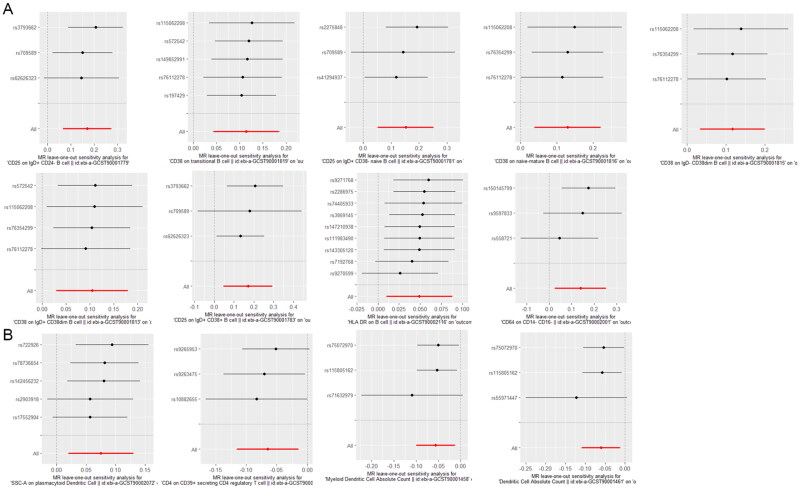
Forest Plots of causal relationship between immune cell and AKI in the results of ‘leave-one-out’ analysis in the forward analysis. (A) Immunophenotypes on AFR. (B) Immunophenotypes on rapid kidney function decline.

**Figure 5.**
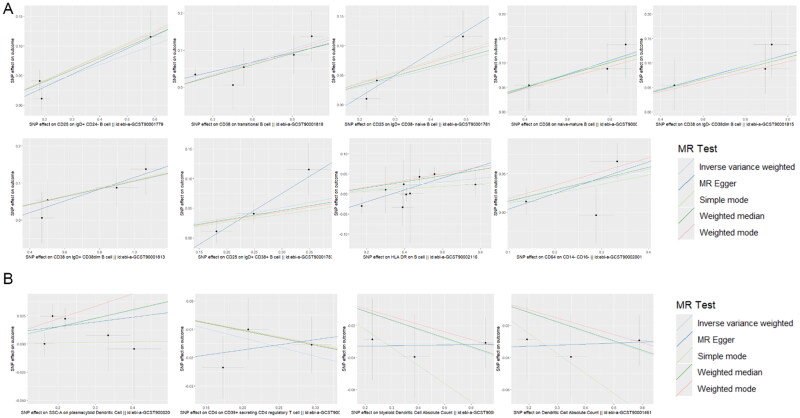
Estimates of immune cell effects across different Mendelian randomization models for AKI. (A) Immunophenotypes on AFR. (B) Immunophenotypes on rapid kidney function decline.

Sensitivity analyses, including the MR-Egger regression, weighted median, and leave-one-out approaches, indicated no evidence of horizontal pleiotropy or significant heterogeneity among the instrumental variables (Table 2). Cochran’s Q test further confirmed the homogeneity of the results, supporting the reliability of the identified associations. Furthermore, the ‘leave-one-out’ analysis demonstrated the robustness of our MR analysis, as it was not influenced by any individual SNP (Figure 4). The scatter plot of SNP effect estimates from different models is shown in Figure 5. The fitted curves of the five models exhibit a similar overall direction, with most models having relatively consistent slopes. The intercept of the IVW model is close to zero.

**Table 2.** Heterogeneity and horizontal pleiotropy of positive results of MR analysis with immune cells as exposure and ARF and rapid kidney function declineas as outcome.

Traits	Heterogeneity		Pleiotropy		
	Q	Q_pval	Egger_intercept	SE	pval
CD25 on IgD + CD24- B cell	1.515	0.469	−0.013	0.033	0.764
CD38 on transitional B cell	1.280	0.865	0.017	0.025	0.536
CD25 on IgD + CD38- naive B cell	2.220	0.330	−0.061	0.047	0.419
CD38 on naive-mature B cell	0.202	0.903	−0.007	0.113	0.958
CD38 on IgD- CD38dim B cell	0.291	0.865	−0.003	0.115	0.984
CD38 on IgD + CD38dim B cell	0.545	0.909	−0.043	0.093	0.688
CD25 on IgD + CD38+ B cell	2.576	0.276	−0.097	0.063	0.365
HLA DR on B cell	9.248	0.322	−0.050	0.025	0.085
CD64 on CD14- CD16-	2.267	0.322	−0.016	0.047	0.787
SSC-A on plasmacytoid Dendritic Cell	3.748	0.441	0.007	0.030	0.835
CD4 on CD39+ secreting CD4 regulatory T cell	1.452	0.484	−0.025	0.028	0.537
Myeloid Dendritic Cell Absolute Count	0.998	0.607	−0.033	−0.033	0.518
Dendritic Cell Absolute Count	1.101	0.577	−0.034	0.034	0.502

## Discussion

Based on a large amount of publicly available genetic data, we investigated the causal relationship between 731 immune cell phenotypes and AKI at the genetic level. To our knowledge, this is the first MR and co-localization analysis to explore the causal relationship between multiple immune phenotypes and ARF and rapid kidney function decline. Our study identified 9 immune phenotypes associated with ARF and 4 immune phenotypes associated with rapid kidney function decline. The findings add to the growing body of evidence that immune dysregulation is central to the pathogenesis of AKI, and they suggest several novel immune cell biomarkers and potential therapeutic targets.

B cells are essential components of humoral immunity, responsible for antigen presentation and cytokine secretion, both of which influence the activation of T cells and the broader immune response [5]. Our study revealed that B cell phenotypes, particularly those expressing CD25 and CD38, were significantly associated with an increased risk of ARF. CD25, as part of the interleukin-2 (IL-2) receptor, is crucial for regulating immune responses [20]. A clinical study has demonstrated a significant increase in serum CD25 levels in patients with septic AKI, suggesting its potential as a novel biomarker for the progression of septic AKI [21]. Given this evidence, CD25-positive B cells may serve as a target for early diagnosis and risk stratification in AKI patients. Future clinical applications could involve routine monitoring of CD25 expression in peripheral blood to identify high-risk patients, allowing for earlier intervention strategies such as immunomodulatory therapies targeting IL-2 signaling. The positive correlation between CD25-positive B cells and ARF suggests that hyperactivation of B cells, driven in part by IL-2 signaling, may contribute to kidney injury through inflammatory pathways. This aligns with previous studies linking IL-2 signaling to B cell-mediated immune responses that can exacerbate tissue damage [22]. Therapeutically, IL-2 blockade or modulation strategies, including low-dose IL-2 therapy aimed at selectively expanding regulatory T cells (Tregs) while suppressing pro-inflammatory B cell responses, could be explored as potential interventions in AKI. Future research should focus on preclinical models to assess the efficacy of IL-2-targeted therapies in reducing kidney injury and inflammation. Clinical trials evaluating the safety and effectiveness of these approaches in AKI patients would further clarify their translational potential.

In addition, CD38, an ectoenzyme involved in cellular adhesion, signal transduction, and calcium signaling, was also strongly associated with ARF. Specifically, transitional B cells and naïve-mature B cells expressing CD38 were positively correlated with ARF risk. This finding supports the hypothesis that CD38-induced B cell activation exacerbates kidney injury by promoting pro-inflammatory responses [23,24]. The role of CD38 in autoimmune and inflammatory diseases further corroborates its potential contribution to AKI pathogenesis. Given the established role of CD38 in autoimmune and inflammatory diseases, targeting CD38-expressing B cells could represent a promising therapeutic approach. Inhibitors of CD38, such as daratumumab, have been successfully used in hematological malignancies and may have potential in modulating B cell-driven inflammation in AKI [25,26]. Future studies should explore the feasibility of repurposing CD38 inhibitors for AKI treatment, initially in preclinical models and subsequently in clinical trials focusing on immune-mediated kidney injury.

In addition to B cells, monocytes expressing CD64 were also found to be positively associated with ARF. CD64 is a high-affinity receptor for IgG, and its expression is indicative of monocyte activation. The involvement of CD64+ monocytes suggests that these cells may contribute to kidney injury through mechanisms such as antibody-dependent cellular cytotoxicity and the release of pro-inflammatory cytokines [27]. This highlights the potential for therapeutic strategies that modulate monocyte activity to reduce inflammation and protect renal function in AKI patients. Additionally, measuring CD64 expression in circulating monocytes may serve as a predictive biomarker for AKI progression, aiding in early diagnosis and patient stratification.

Dendritic cells (DCs) are critical for initiating immune responses and maintaining tolerance. Our study demonstrated a positive correlation between SSC-A on plasmacytoid dendritic cells (pDCs) and rapid kidney function decline. pDCs are known for their ability to produce large amounts of type I interferons in response to pathogens, which can lead to inflammation [28]. The observed association between pDCs and rapid kidney function decline suggests that pDC-derived interferons play a key role in exacerbating renal injury by promoting inflammatory cytokine production. This is consistent with previous studies highlighting the inflammatory role of pDCs in tissue damage [29].Given the potential contribution of pDCs to kidney injury, therapeutic strategies targeting pDC activation or interferon signaling could be explored. For instance, interferon-blocking antibodies or pDC depletion strategies could help mitigate inflammatory damage in AKI patients. Future studies should assess the efficacy of such interventions in preclinical AKI models before considering their translation into clinical trials.

Conversely, we found a negative correlation between myeloid dendritic cell (mDC) counts and rapid kidney function decline, which suggests that mDCs may have a protective role. mDCs are capable of inducing regulatory T cells (Tregs) and producing anti-inflammatory cytokines that help control inflammation [30]. Our findings support the hypothesis that mDCs contribute to kidney protection in AKI by fostering an anti-inflammatory environment. This protective function of mDCs has been demonstrated in other contexts of immune regulation [31].

Regulatory T cells (Tregs) are central to maintaining immune homeostasis and preventing excessive inflammation. Our study found that CD39+ Tregs, specifically those expressing CD4, were negatively associated with rapid kidney function decline. CD39, an ectonucleotidase expressed on Tregs, catalyzes the conversion of ATP and ADP to AMP, which is further converted to adenosine—a potent immunosuppressive molecule [32]. The negative association between CD39+ Tregs and kidney function decline suggests that these cells exert a renoprotective effect, possibly by reducing inflammation and promoting tissue healing. Previous studies have highlighted the role of CD39-mediated adenosine production by Tregs in controlling immune responses and preventing tissue damage [33]. Enhancing Treg function or CD39 activity could serve as a promising strategy for AKI treatment. Potential therapeutic approaches include low-dose IL-2 therapy to selectively expand Tregs or the use of adenosine analogs to mimic CD39-mediated immunosuppression. Future studies should explore the feasibility of these approaches in AKI models and evaluate their safety and efficacy in clinical trials.

As an emerging biomarker, immune phenotyping holds significant promise for early diagnosis and personalized treatment of AKI. Compared to traditional biomarkers, such as serum creatinine and urine output, immune phenotypes may provide a more sensitive and specific reflection of early renal damage, especially in the initial stages of the disease [34]. By identifying specific immune cell subsets and their alterations, clinicians could better identify high-risk patients and implement timely interventions to mitigate kidney injury and improve outcomes. Our findings support the clinical application of immune phenotyping as a diagnostic tool for AKI. In particular, immune cell subset changes could serve as early indicators of AKI before significant renal dysfunction is detectable through conventional biomarkers. To translate immune phenotyping into clinical practice, standardized protocols for immune cell profiling in AKI patients should be developed. This could include flow cytometry-based assays or single-cell RNA sequencing approaches integrated into routine clinical workflows. Additionally, combining immune phenotyping with existing clinical risk assessment tools, such as the APACHE II and SOFA scores, could enhance early detection of AKI and enable more accurate risk stratification. A stepwise validation process should be undertaken, beginning with large-scale observational studies to confirm the predictive value of immune markers, followed by prospective interventional trials evaluating immune-based therapeutic strategies. Although our study provides preliminary evidence of the immune phenotype-AKI causal relationship, further validation through larger cohorts and functional studies is required before these findings can be applied to clinical practice. Future efforts should focus on refining immune-based diagnostic and therapeutic approaches, with the ultimate goal of integrating personalized immunomodulatory strategies into AKI management.

The use of MR in this study provided a powerful tool to infer causal relationships between immune phenotypes and AKI, addressing some of the key limitations of observational studies, such as confounding and reverse causality. By utilizing genetic variants as instrumental variables, we reduced the risk of confounding that often complicates traditional observational designs. However, it is important to acknowledge several limitations of this approach. Firstly, while our study primarily focuses on individuals of European ancestry, we recognize that this focus may limit the generalizability of our findings to other ethnic groups. Immunological traits and their genetic underpinnings can vary significantly across different populations, which could lead to divergent associations with AKI. Consequently, our findings may not fully capture these inter-population variations. To enhance the generalizability and applicability of these results, future research should incorporate diverse populations, particularly those of Asian and African descent. This would provide a more comprehensive understanding of the role of immune phenotypes in AKI and potentially lead to more tailored therapeutic strategies that address the needs of diverse patient populations. Secondly, while summary-level data offer valuable insights into genetic associations, they lack the resolution required to explore the underlying biological mechanisms at a cellular or molecular level. These data, though powerful in terms of statistical power due to their large sample sizes, are limited in their ability to investigate specific biological pathways and causal mechanisms. To overcome this limitation, it is crucial to functionally validate these associations through *in vitro* and *in vivo* experiments. For example, specific immune cell types can be isolated using flow cytometry and co-cultured with renal tubular epithelial cells to confirm the biological relevance of these genetic findings. Such studies could provide direct evidence of causality and help elucidate the precise role of immune phenotypes in the pathophysiology of AKI. In conclusion, while our study provides strong evidence of causal links between immune phenotypes and AKI, further research is required to validate these associations in more ethnically diverse populations and to explore the molecular pathways that underlie these relationships. This will help ensure that the findings are broadly applicable and lead to actionable insights for improving patient outcomes in AKI.

## Conclusion

In summary, this study provides novel insights into the causal relationship between immune cell phenotypes and AKI using a Mendelian randomization approach. The findings suggest that specific immune cells, including activated B cells, plasmacytoid dendritic cells, and regulatory T cells, play critical roles in the development and progression of AKI. These immune phenotypes may serve as potential biomarkers and therapeutic targets for the prevention and management of AKI. Future research should focus on validating these findings in clinical settings and elucidating the underlying biological mechanisms, ultimately contributing to improved outcomes for patients with AKI.

## Supplementary Material

Figure 4B.tif

Figure 1.tif

Figure 3.tif

Figure 5B.tif

Figure 5A.tif

Figure 4A.tif

Supplementary document 1.xlsx

Figure 2.tif

## Data Availability

The authors will provide the original data supporting the conclusions of this study without reservation. For further inquiries, please get in touch with the corresponding author directly.
