# Supplementary figures and images for: The association between immune cells and acute kidney injury: insights from Mendelian randomization
Source: Ren Fail. 2025 Mar 2;47(1):2471011. doi: 10.1080/0886022X.2025.2471011 (PMC11878167; doi:10.1080/0886022X.2025.2471011)

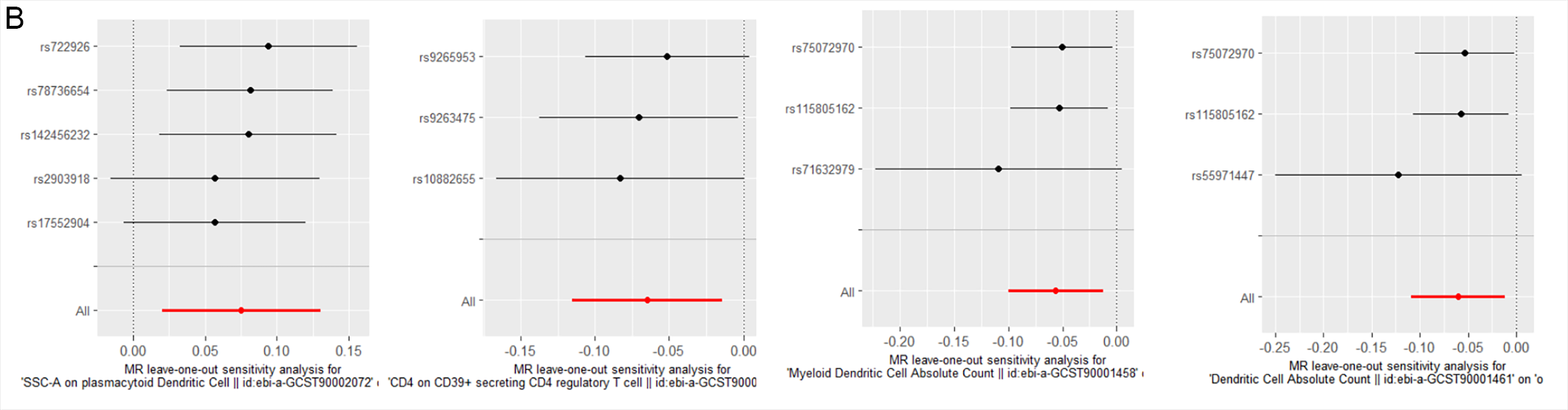

Supplement: Figure 4B.tif [file IRNF_A_2471011_SM6077.tif]

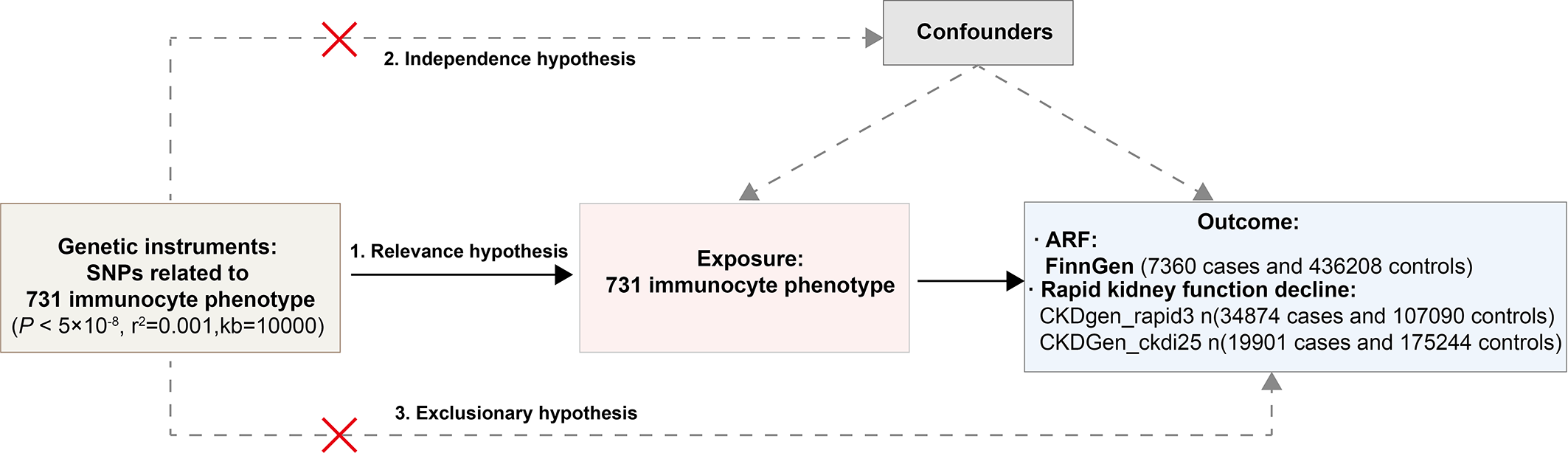

Supplement: Figure 1.tif [file IRNF_A_2471011_SM6076.tif]

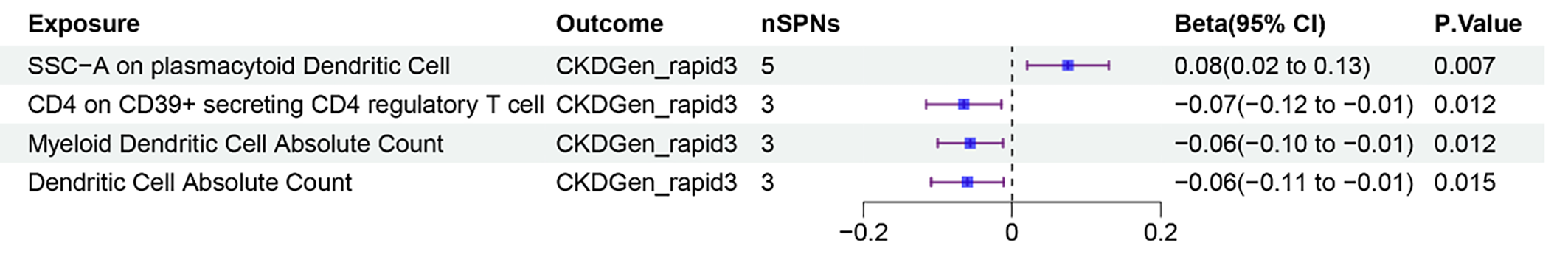

Supplement: Figure 3.tif [file IRNF_A_2471011_SM6075.tif]

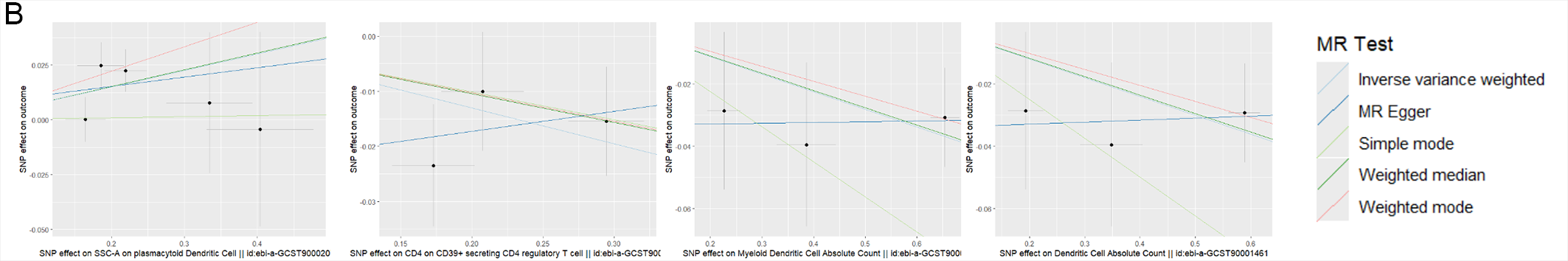

Supplement: Figure 5B.tif [file IRNF_A_2471011_SM6074.tif]

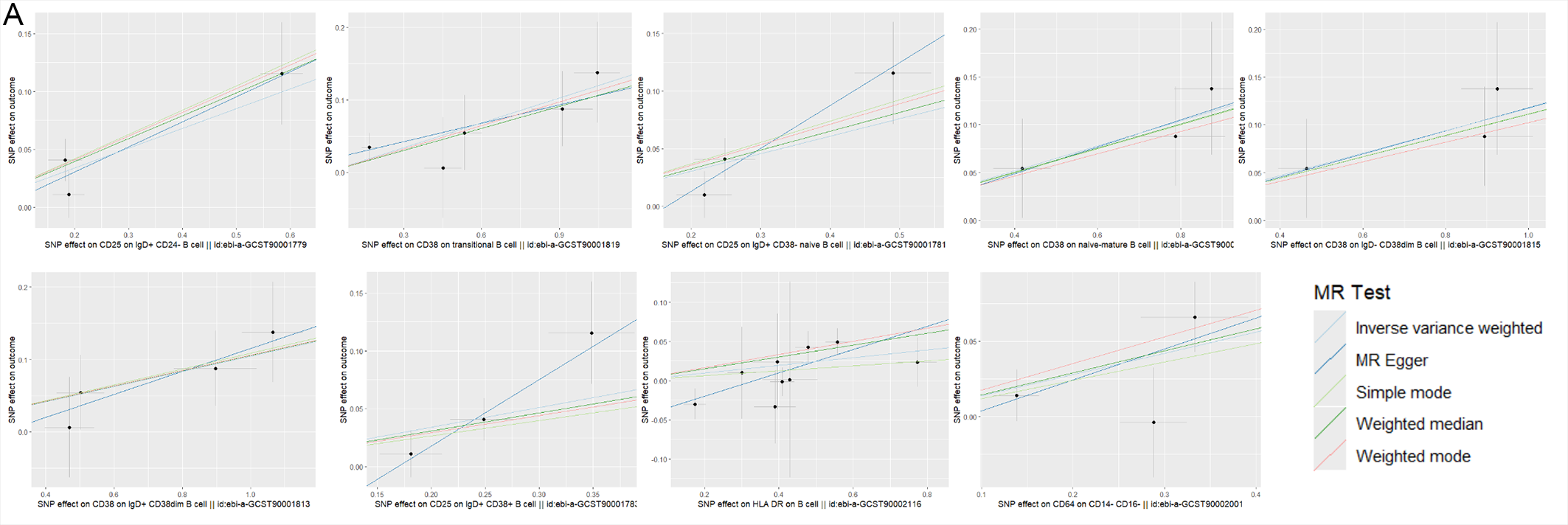

Supplement: Figure 5A.tif [file IRNF_A_2471011_SM6073.tif]

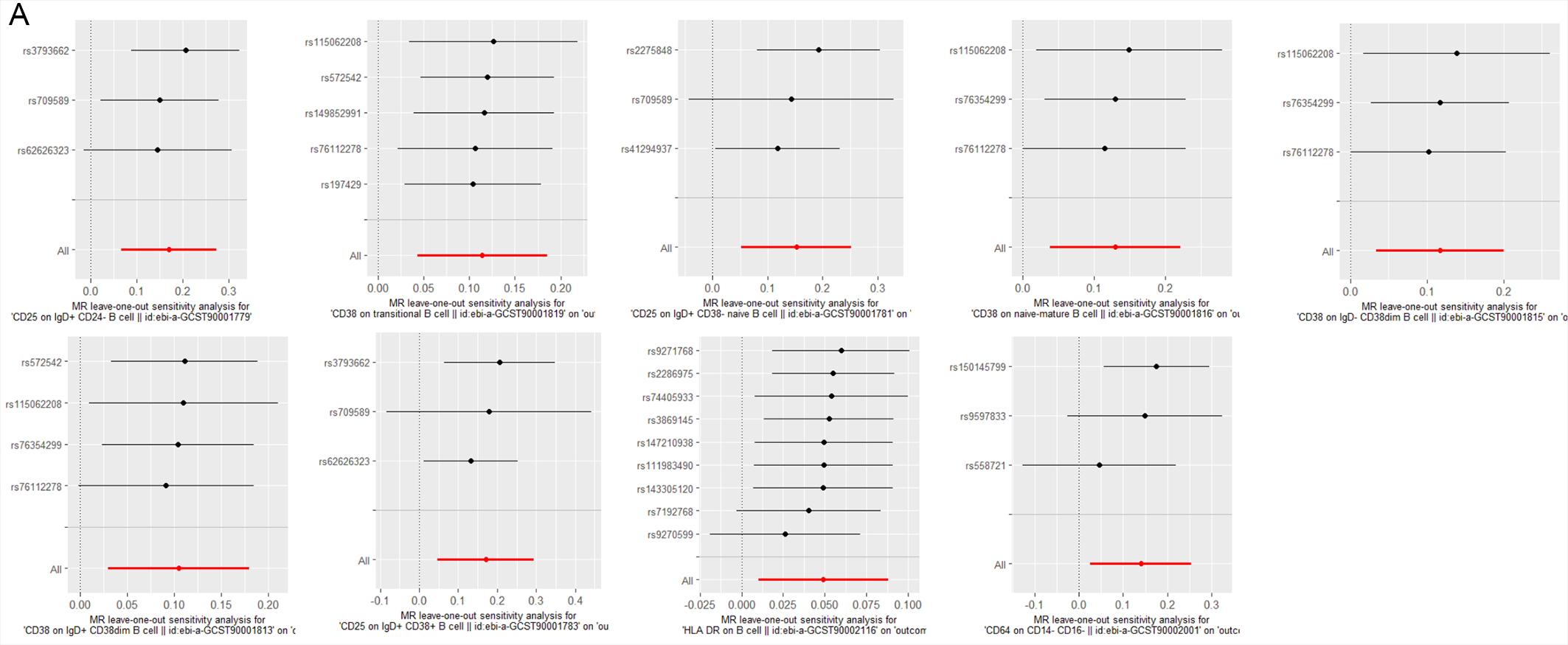

Supplement: Figure 4A.tif [file IRNF_A_2471011_SM6072.tif]

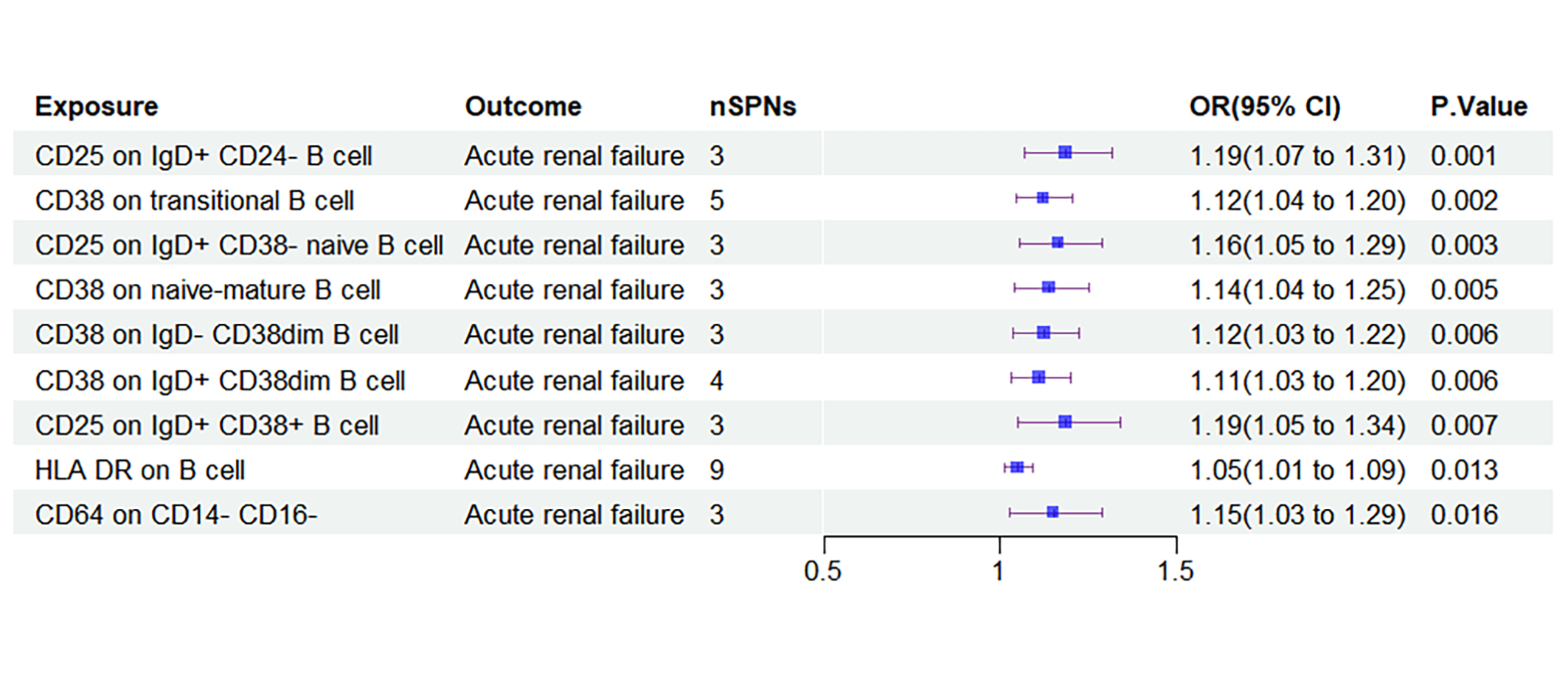

Supplement: Figure 2.tif [file IRNF_A_2471011_SM6070.tif]
